# Supplementary figures and images for: 1,8 Cineole and Ellagic acid inhibit hepatocarcinogenesis via upregulation of MiR-122 and suppression of TGF-β1, FSCN1, Vimentin, VEGF, and MMP-9
Source: PLoS One. 2022 Jan 26;17(1):e0258998. doi: 10.1371/journal.pone.0258998 (PMC8791452; doi:10.1371/journal.pone.0258998)

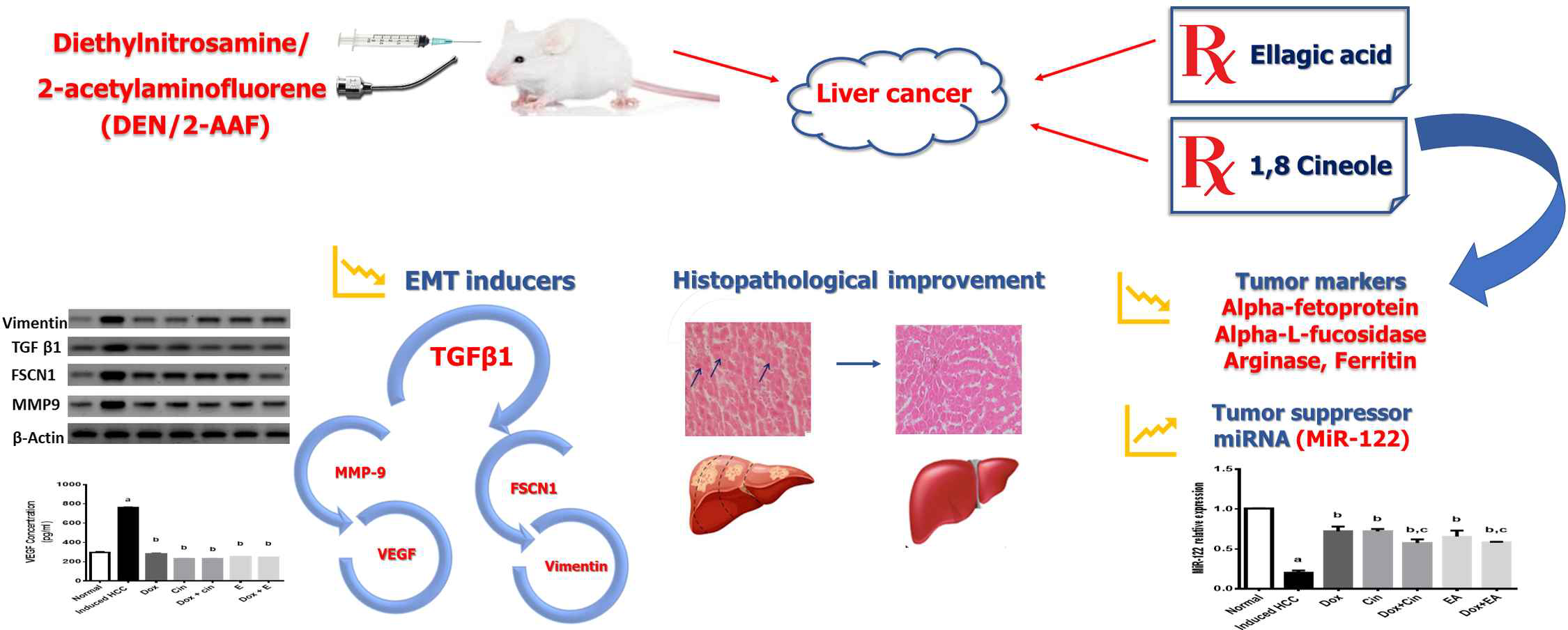

Supplement: S1 Graphical abstract — (TIF) [file pone.0258998.s001.tif]

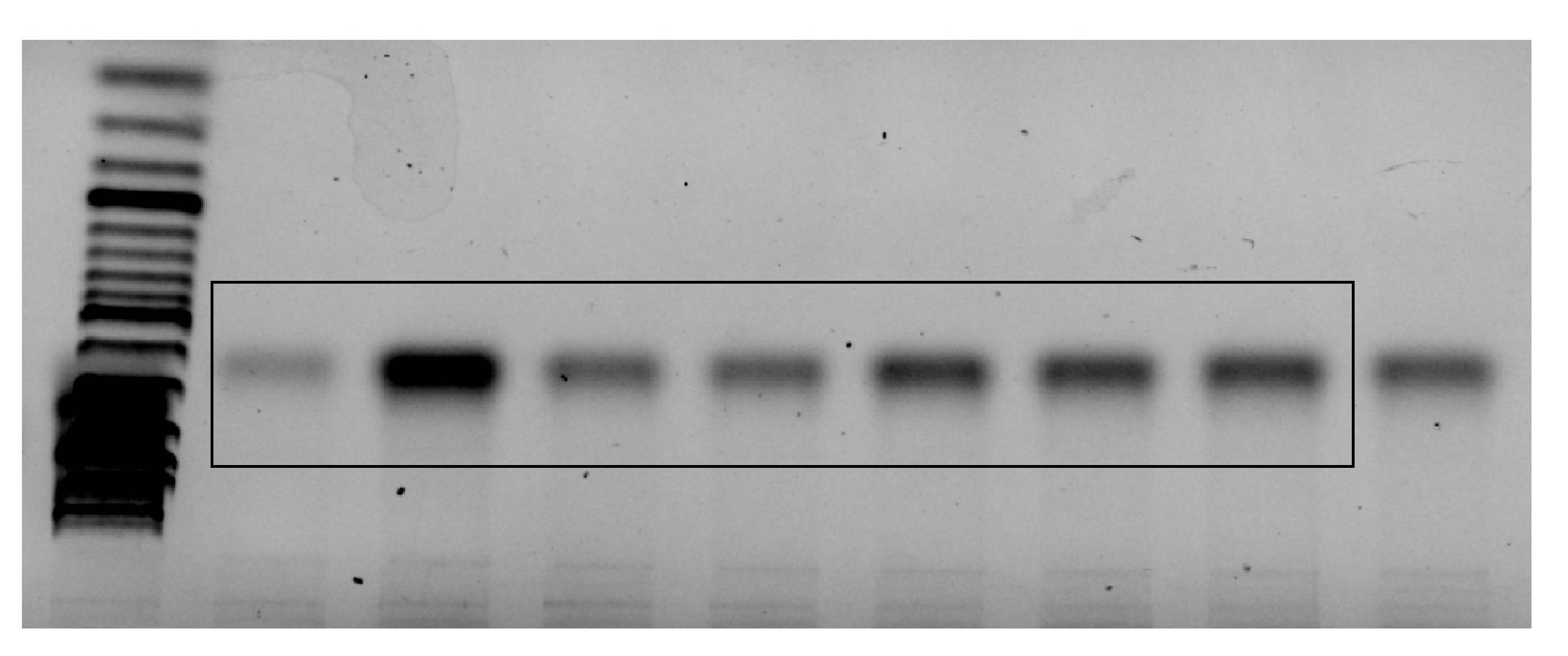

Supplement: S1 Raw images — (ZIP) [file pone.0258998.s002.zip › Vimentin.jpg]

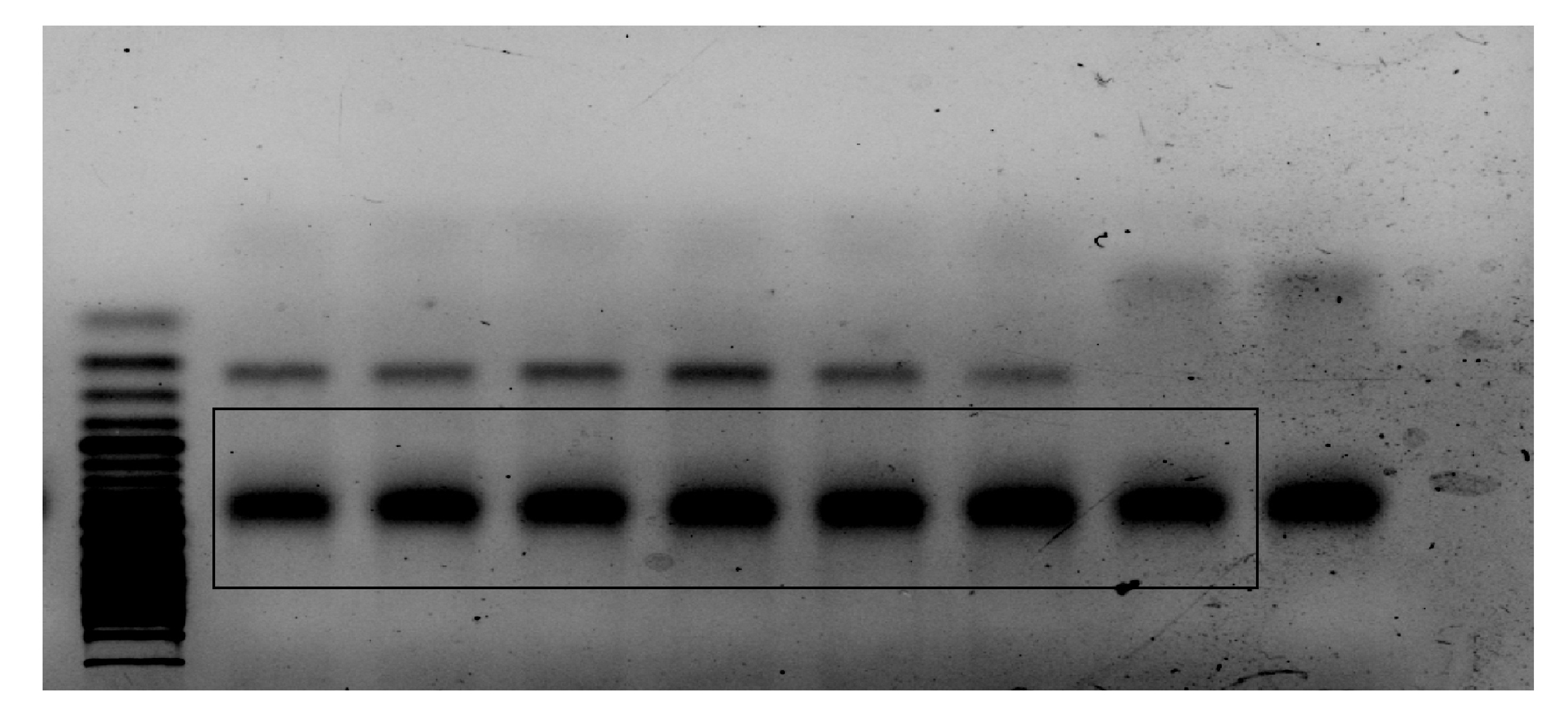

Supplement: S1 Raw images — (ZIP) [file pone.0258998.s002.zip › beta actin.jpg]

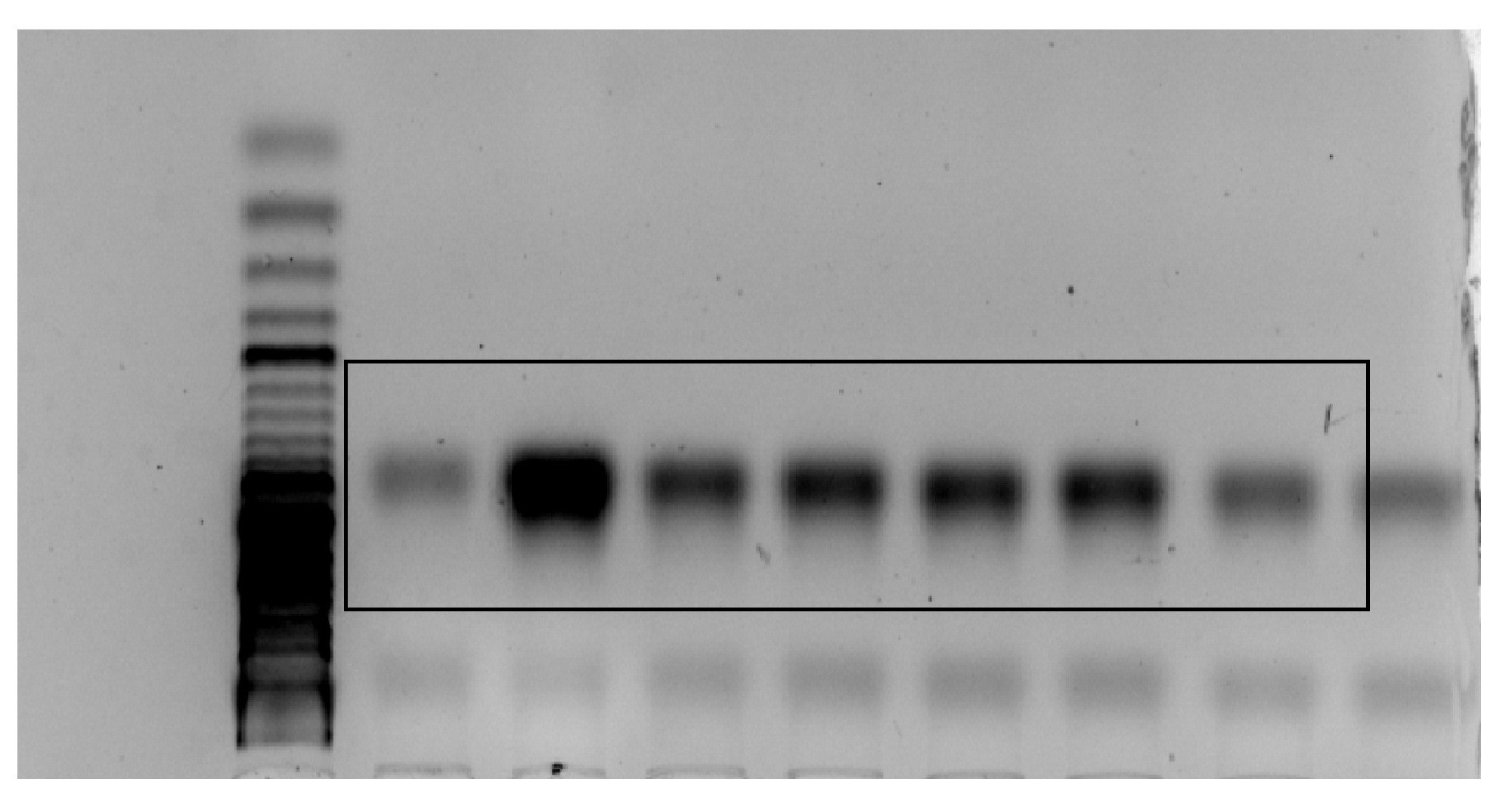

Supplement: S1 Raw images — (ZIP) [file pone.0258998.s002.zip › FSCN1.jpg]

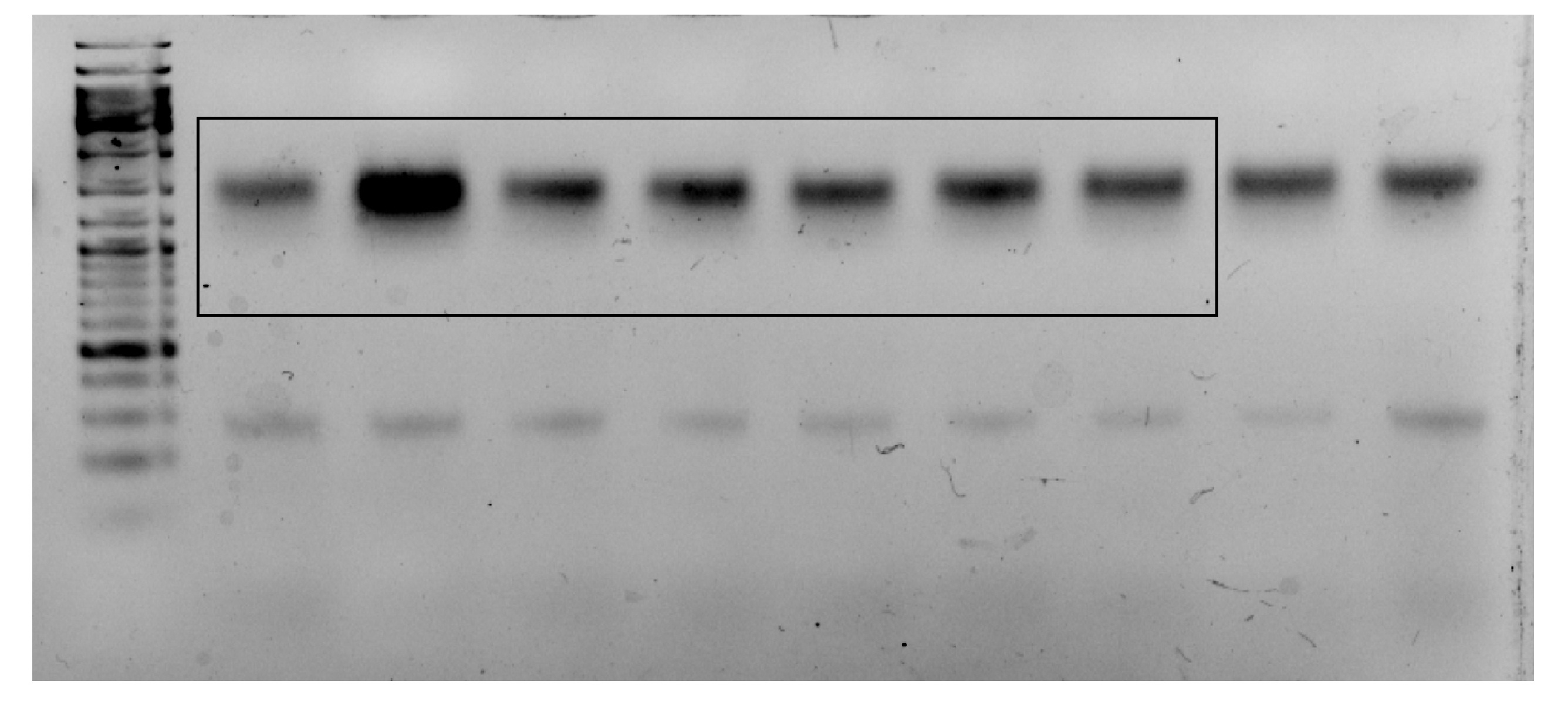

Supplement: S1 Raw images — (ZIP) [file pone.0258998.s002.zip › MMP-9.jpg]

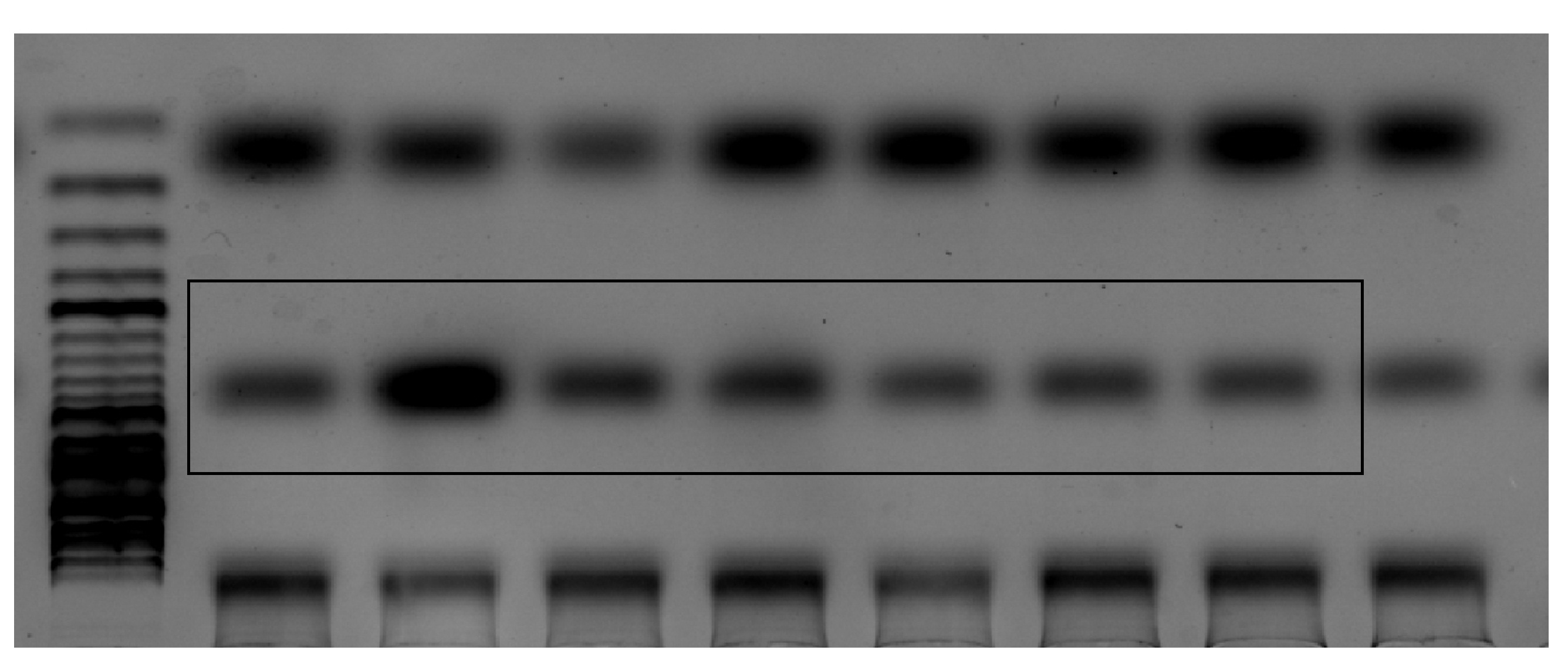

Supplement: S1 Raw images — (ZIP) [file pone.0258998.s002.zip › TGF beta 1.jpg]
