# Supplementary material for: 1,8 Cineole and Ellagic acid inhibit hepatocarcinogenesis via upregulation of MiR-122 and suppression of TGF-β1, FSCN1, Vimentin, VEGF, and MMP-9
Source: PLoS One. 2022 Jan 26;17(1):e0258998. doi: 10.1371/journal.pone.0258998 (PMC8791452; doi:10.1371/journal.pone.0258998)
Supplement: Attachment — Submitted filename: Response to Reviewers.docx [file pone.0258998.s003.docx]

**Additional Editor Comments:**

- Well performed and well written manuscript. But language is the major issue and i suggest to send the manuscript for language editing company for correction.

**Response:** We appreciated the editor's comment. Accordingly, we sent the manuscript to a language editing company and the proof was attached.

**Journal Requirements:**

**1.** Please ensure that your manuscript meets PLOS ONE's style requirements, including those for file naming. The PLOS ONE style templates can be found at

https://journals.plos.org/plosone/s/file?id=wjVg/PLOSOne_formatting_sample_main_body.pdf and

<https://journals.plos.org/plosone/s/file?id=ba62/PLOSOne_formatting_sample_title_authors_affiliations.pdf>

**Response:** We respect the journal requirements, and the corrections have been done.

**2.** Please review your reference list to ensure that it is complete and correct. If you have cited papers that have been retracted, please include the rationale for doing so in the manuscript text, or remove these references and replace them with relevant current references. Any changes to the reference list should be mentioned in the rebuttal letter that accompanies your revised manuscript. If you need to cite a retracted article, indicate the article’s retracted status in the References list and also include a citation and full reference for the retraction notice.

**Response:** We appreciated the reviewer's comment. We revised the whole references list and added four references to be a final of 60. All the changes have been done to meet the journal requirement.

The added references are listed below:

1. Ferlay J, Colombet M, Soerjomataram I, Mathers C, Parkin DM, Pineros M, et al. Estimating the global cancer incidence and mortality in 2018: GLOBOCAN sources and methods. Int J Cancer. 2019;144(8):1941-53. Epub 2018/10/24. doi: 10.1002/ijc.31937. PubMed PMID: 30350310
2. Sobreira Dantas Nobrega de Figueiredo FR, Monteiro AB, Alencar de Menezes IR, Sales VDS, Peticia do Nascimento E, Kelly de Souza Rodrigues C, et al. Effects of the Hyptis martiusii Benth. leaf essential oil and 1,8-cineole (eucalyptol) on the central nervous system of mice. Food Chem Toxicol. 2019;133:110802. Epub 2019/09/08. doi: 10.1016/j.fct.2019.110802. PubMed PMID: 31493462.
3. Abdel-Hamid NM, Abass SA, Mohamed AA, Muneam Hamid D. Herbal management of hepatocellular carcinoma through cutting the pathways of the common risk factors. Biomed Pharmacother. 2018;107:1246-58. Epub 2018/09/28. doi: 10.1016/j.biopha.2018.08.104. PubMed PMID: 30257339
4. Choudhari AS, Mandave PC, Deshpande M, Ranjekar P, Prakash O. Phytochemicals in Cancer Treatment: From Preclinical Studies to Clinical Practice. Front Pharmacol. 2019;10:1614. Epub 2020/03/03. doi: 10.3389/fphar.2019.01614. PubMed PMID: 32116665

**3.** In your Methods section, please provide additional information on the animal research and ensure you have included details on : (1) how often the animal health was monitored (2) whether any animals died during induction of hepatocarcinogenesis, and if so, how many were found dead? (3) specific criteria of how animal health was monitored.

**Response:** We are thankful for the reviewer's accuracy and in order to respond to the reviewer comment, we added the specific criteria of animal health and the number of dead animals during induction of hepatocarcinogenesis in the method section.

**(1)** The general health conditions of animals concerning food consumption, water intake, and body weight were observed during all the experimental time besides signs of distress including respiratory changes, body temperature, or even unusual behavior were also observed. We did not notice a significant variation in the previous parameters between the experimental groups except for the DEN/2-AAF group that showed a modest weight loss compared with the others **(Page 5, lines from 100 to 105)**.

**(2)** Throughout the initiation phase of DEN treatment (first eight weeks), only one rat was dead. After starting 2-AAF administration (promotion phase), another two rats from the DEN/2-AAF induced HCC group died **(Page 5, lines 111-113).**

**4.** PLOS ONE now requires that authors provide the original uncropped and unadjusted images underlying all blot or gel results reported in a submission’s figures or Supporting Information files. This policy and the journal’s other requirements for blot/gel reporting and figure preparation are described in detail at https://journals.plos.org/plosone/s/figures#loc-blot-and-gel-reporting-requirements and https://journals.plos.org/plosone/s/figures#loc-preparing-figures-from-image-files. When you submit your revised manuscript, please ensure that your figures adhere fully to these guidelines and provide the original underlying images for all blot or gel data reported in your submission. See the following link for instructions on providing the original image data: https://journals.plos.org/plosone/s/figures#loc-original-images-for-blots-and-gels.

In your cover letter, please note whether your blot/gel image data are in Supporting Information or posted at a public data repository, provide the repository URL if relevant, and provide specific details as to which raw blot/gel images, if any, are not available. Email us at plosone@plos.org if you have any questions.

**Response:** We understand the journal requirement and accordingly we uploaded a separate PDF file with the raw data of the original uncropped blot images.

5. Your ethics statement should only appear in the Methods section of your manuscript. If your ethics statement is written in any section besides the Methods, please delete it from any other section.

**Response:** We respect the reviewer's comment. So, the ethics statement is only now in the method section.
